# Radial Hadamard-encoded ^19^F-MRI

**DOI:** 10.1007/s10334-025-01254-2

**Published:** 2025-05-30

**Authors:** Kian Tadjalli Mehr, Johannes Fischer, Felix Spreter, Simon Reiss, David Boll, Ali Caglar Özen, Deepa Gunashekar, Constantin von zur Mühlen, Alexander Maier, Michael Bock

**Affiliations:** 1 Division of Medical Physics, Department of Diagnostic and Interventional Radiology, Faculty of Medicine, University Medical Center Freiburg, University of Freiburg, Killianstr. 5a, 79106 Freiburg, Germany; 2 Department of Cardiology, Faculty of Medicine, University Medical Center Freiburg, University of Freiburg, Freiburg, Germany

**Keywords:** Magnetic resonance imaging, Perfluorocarbons, Fluorine-19 magnetic resonance imaging

## Abstract

**Objectives:**

Developing a ^19^F imaging method to acquire images of the molecular inflammation tracer perfluorooctyl bromide (PFOB) without chemical shift artifacts.

**Materials and methods:**

PFOB is a molecular tracer that can be used to track the response of myeloid cells. However, imaging of PFOB with ^19^F-MRI is challenging due to its complex spectrum which leads to unwanted chemical shift artifacts. Spectral HE allows for separate reconstructions of each peak of the PFOB spectrum, which was combined into a single image after resonance shift correction. In this work, a Hadamard-encoded (HE) radial 3D UTE sequence was tested in phantoms and in vivo in a pig, measuring the ^19^F signal in the spleen at different times after injection.

**Results:**

Chemical shift artifacts were effectively suppressed with HE, and an SNR > 100 was observed for the ^19^F signal in the spleen 2 days after injection. The signal decreased over time, and 7 days after injection it was reduced by 30%.

**Discussion:**

Chemical shift artifact correction using HE allowed for in vivo ^19^F PFOB imaging of labeled monocytes with a high SNR. Compared to spectrally selective excitation, HE increased the PFOB ^19^F-MRI signal by 10%, and the simple HE-algorithm could be directly integrated into the image reconstruction of the MRI system.

## Introduction

Novel therapeutic approaches to treat inflammatory diseases make molecular inflammation imaging an attractive therapeutic tool [1]. Myeloid cells are involved in inflammatory healing processes. They accumulate in the spleen, where they remain silent until activation in response to inflammatory events such as myocardial infarction (MI), stroke, or others. These cells can be labeled using biologically inert perfluorocarbons (PFCs) that are injected into the blood stream [2]. The distribution of the PFC-labeled cells can be imaged with ^19^F-MRI [3], which makes PFCs an excellent imaging marker for inflammation due to the absence of other ^19^F-MRI background signals [4, 5]. Fluorine-19 has a natural abundance of nearly 100% and a gyromagnetic ratio of 40.05 MHz/T leading to a high MR sensitivity of 83% of that of protons [6]; however, ^19^F-MRI of PFCs is limited by the low millimolar concentrations of ^19^F in an in vivo application.

Due to the background-free ^19^F-MRI signal, the concentration of inflammatory cells and, in turn, the inflammatory reaction can be directly quantified from the ^19^F-MRI signal of the PFCs [7]. Several different PFCs have been proposed for this application [8–10]. In this work, we use perfluorooctyl bromide (PFOB), which has been used in clinical trials before and has a relatively low biological half-life of about 12 days [8]. Unlike some other PFCs, PFOB is known to exhibit significant chemical shift artifacts in ^19^F-MRI due to its complex ^19^F NMR-spectrum. One approach to mitigate chemical shift artifacts is the selective excitation of single resonances of the spectrum at the cost of an overall reduced signal intensity. Previous works have performed cellular ^19^F imaging of PFOB in this way [11, 12]. Alternatively, all resonances can be excited and the chemical shift artifacts can be removed during data post-processing using a deconvolution approach [13–15], using phase information at different echo times [16, 17] or ^19^F-MRI data can be acquired with two different directions of the readout direction leading to a characteristic change of the chemical shift artifact patterns which can be exploited to suppress them [18].

In this work, we use a different concept for chemical shift correction which is based on spectral Hadamard-encoding (HE) [19, 20]. HE is combined with radial data acquisition based on an ultra-short TE (UTE) sequence, and the method is tested in vivo in pigs with ^19^F-MRI of the spleen.

## Materials and methods

### Perfluorooctyl bromide (PFOB)

**Fig. 1:**
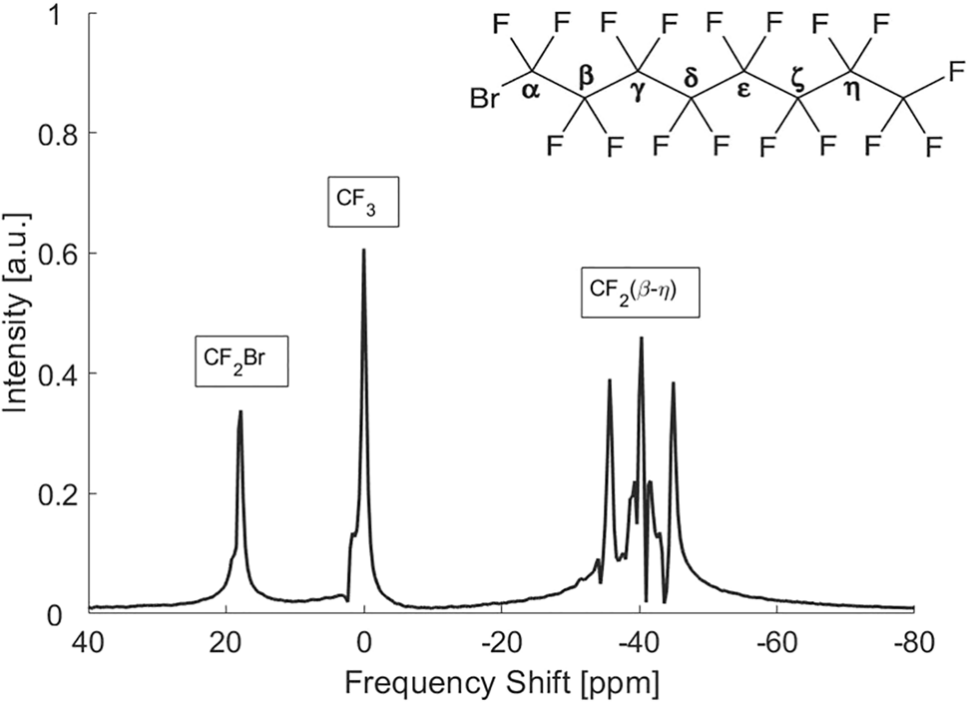
Chemical structure and chemical shift spectrum of PFOB at 3 T

Perflubron or PFOB is a perfluorinated derivative of the hydrocarbon octane, where all 18 hydrogen atoms have been replaced with 17 fluorine (^19^F) and 1 bromine (Br) atoms leading to 1 terminal CF_3_ group, 1 terminal CF_2_Br group, and 6 CF_2_ groups (Fig. 1). In the ^19^F MR spectrum, these groups have different chemical shifts of ${\delta }_{{\text{CF}}_{2}\text{Br}}=+18.1 \text{ ppm}$ and ${\delta }_{{\text{CF}}_{2}}=-40.1 \text{ ppm}$ relative to the central CF_3_ resonance (here set to ${\delta }_{{\text{CF}}_{3}}=0 \text{ ppm}$) [8, 21]. The terminal Br atom breaks the structural symmetry of the molecule and gives rise to small variations in chemical shift of the CF_2_ resonances ranging from $-35.7 \text{ ppm}$ for the closest CF_2_ group, *β*, to the most distant group *η* at $-44.8 \text{ ppm}$. In this work, these resonances cannot be resolved so that we consider only three distinct resonances.

### Hadamard-encoding scheme

In MRI with conventional Cartesian encoding, chemical shift artifacts manifest as ghost images of the different resonances that appear spatially shifted along the readout direction by

1 $$\Delta x=\delta \cdot \frac{\gamma {B}_{0}}{BW},$$

where BW is the receiver bandwidth and $\delta$ is the chemical shift of the resonance. The most common occurrence of chemical shift correction is the fat–water shift in ^1^H MRI. Many methods were developed to remove the chemical shift artifacts by suppressing the fat signal [22]. However, in ^19^F-MRI, the tracer concentration is low, and signal from all resonances is desired to improve SNR. Thus, rather than using suppression techniques to avoid chemical shift artifacts, we propose a spectral encoding/decoding scheme that allows for the simultaneous acquisition of signal from all resonances.

HE has been previously applied in MRI, for example, to counteract aliasing artifacts [23], or in simultaneous multislice acquisitions [24]. The general concept is to encode a vector of information by multiplying it with a matrix of orthogonal column vectors, which can later be decoded by multiplication with the transposed matrix. Here, we use a Hadamard matrix $\mathcal{H}$, to encode the signal from each separate resonance, by acquiring the same k-space data four times with four different RF excitation pulses ${B}_{1}$.

2 $$\left.\left(\begin{array}{c}{B}_{\text{1,1}}\left(t\right)\\ {B}_{\text{1,2}}\left(t\right)\\ {B}_{\text{1,3}}\left(t\right)\\ {B}_{\text{1,4}}\left(t\right)\end{array}\right.\right)=\mathcal{H}{\varvec{A}}=\left(\begin{array}{ccc}1& 1& 1\\ 1& -1& -1\\ 1& 1& -1\\ 1& -1& 1\end{array}\right)\cdot \left(\begin{array}{c}{B}_{1, \mu }\left(t\right)\cdot \text{exp}\left(-i{\delta \omega }_{1}t\right)\\ {B}_{1,\nu }\left(t\right)\\ {B}_{1,\xi }\left(t\right)\cdot \text{exp}\left(-i{\delta \omega }_{2}t\right)\end{array}\right),$$

where ${B}_{1,\mu }$ is the RF-envelope of the CF_2_Br-Peak, ${B}_{1,\nu }$ of the CF_3_-Peak, and ${B}_{1,\xi }$ of the CF_2_-Peaks and $\delta {\omega }_{1}=\gamma {B}_{0}{\delta }_{C{F}_{2}Br}$ and $\delta {\omega }_{2}=\gamma {B}_{0}{\delta }_{C{F}_{2}}$ are the off-resonances of the respective resonances relative to the CF_3_ group. Note that the Hadamard matrix is reduced to 4 × 3, because we only need to encode three resonances and the fourth encoding combination is not needed. The frequency of the MRI system is set to the resonance frequency of the CF_3_ resonance. Negative signs in the encoding matrix are effectively 180° phase shifts in the excitation, which means that received signals of the same resonance excited with a negative and a positive sign cancel out after addition of the raw data. While it would be possible to separate the individual CF_2_ resonances as well using a larger Hadamard matrix, this would require longer pulses causing signal loss due to the longer echo time, while only gaining a minimal amount of signal.

**Fig. 2:**
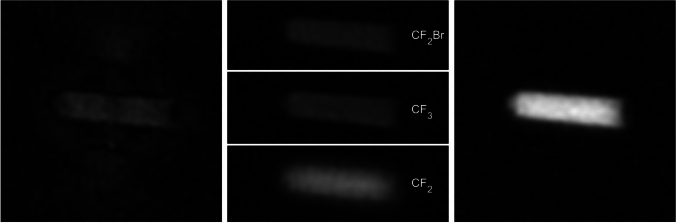
Different steps during the reconstruction process shown with the same windowing. Left: Chemical shift artifacts in a radial sequence, Middle: Reconstructed signals for each peak after Hadamard-decoding. Due to chemical shifts within the CF_2_ multi resonance, this image appears blurred. Right: Combination of all signals via sum of squares

During post-processing, the signal from the three resonances can, therefore, be Hadamard-decoded using the following combinations of signals:

3 $$\left(\begin{array}{c}{S}_{\mu }\\ {S}_{\nu }\\ {S}_{\xi }\end{array}\right)={\mathcal{H}}^{T}{\varvec{S}}=\left(\begin{array}{cccc}1& 1& 1& 1\\ 1& -1& 1& -1\\ 1& -1& -1& 1\end{array}\right)\left.\left(\begin{array}{c}{S}_{1}\\ {S}_{2}\\ {S}_{3}\\ {S}_{4}\end{array}\right.\right),$$

where ${S}_{1},\dots ,{S}_{4}$ are the signals received after each HE pulse, ${B}_{1,i}$. In Eq. (3), the signals from the resonances ${S}_{\mu }, {S}_{\nu },$ or ${S}_{\xi }$ add up constructively, while signals from the other resonances cancel, such that after the four pulses each resonance has been acquired without time loss compared to selective excitation with four averages. After reversing the chemical shift for each of the resonances individually, their signals can be added using a sum of squares combination (Fig. 2). In the ideal case, where all resonances contribute equally, the 3 fluorine atoms from the CF_3_ resonance, the 2 fluorine atoms from the CF_2_Br resonance and the 12 fluorine atoms of the CF_2_ resonances would increase the signal by a factor of $\frac{\sqrt{{12}^{2}+{3}^{2}+{2}^{2}}}{12}=1.04$, over an acquisition with the CF_2_ resonances alone—thus, only a 4% gain would be measurable. In reality, however, the CF_2_-signals dephase quickly and blur due to the uncorrected chemical shifts, which reduces their contribution to the total signal, making it more worthwhile to add the contribution from the other resonances.

### Chemical shift correction

In radial imaging, the readout direction is different for each k-space line (spoke). Thus, artifacts from chemical shifts do not result in a constant spatial offset as in Cartesian imaging, but rather in a blurring of the reconstructed image [15]. Therefore, a chemical shift correction must be performed prior to regridding of the radial data to a Cartesian grid. The evolution of the MR signal phase during data acquisition of an isochromate with chemical shift $\delta$ is given by:

4 $$\varphi \left( {r,{ }t} \right) = \gamma \left( {1 + \delta } \right)\smallint B_{0} + G\left( {t^{\prime } } \right)r{\text{d}}t^{\prime } = \left( {1 + \delta } \right)\gamma B_{0} t + \smallint \gamma G\left( {t^{\prime } } \right)r{\text{d}}t^{\prime } + \smallint \delta \gamma G\left( {t^{\prime } } \right)r{\text{d}}t^{\prime } .$$

The first term describes a linearly increasing phase over time, independent of the spatial location, compared to the resonant signal. The second term is the desired on-resonant phase evolution due to spatial encoding in a magnetic field gradient $G$. The last term is also location dependent, but can be neglected as ${B}_{0}\gg G\cdot r$. The phase evolution of the chemical shift is, therefore, governed by $\delta \gamma {B}_{0}t$, i.e. a linear increase of the phase with time. Thus, after signals of the different resonances are separated using HE, the chemical shift can be corrected by multiplication of each acquired line in k-space with a linearly increasing phase factor $\text{exp}\left(+i\delta \gamma {B}_{0}\kappa \tau \right)$, where $\kappa$ is the index of the pixel within that line and $\tau$ is the dwell time.

### RF pulse shapes

To excite the individual resonances, both Gaussian and sinc shapes were tested as RF-pulses. The Gaussian pulses were defined as

5 $${B}_{1}^{Gauss}\left(t\right)={B}_{1}^{Gauss}\cdot \text{exp}\left(-\frac{{\pi }^{2}{\delta \omega }_{\rm Gauss}^{2}{t}^{2}}{2}\right).$$

Compared to Gauss pulses, sinc pulses can excite a broader range of the spectrum homogeneously. Here, a Hamming-windowed pulse [25] was applied:

6 $${B}_{1}^{sinc}\left(t\right)={B}_{1}^{sinc}\cdot \left(0.54+0.46\cdot \text{cos}\left(\frac{\pi \cdot \delta {\omega }_{\text{sinc}}\cdot t}{6}\right)\right)\cdot \frac{\text{sin}\left(\pi \cdot \delta {\omega }_{\text{sinc}}\cdot t\right)}{\pi \cdot \delta {\omega }_{\text{sinc}}\cdot t},$$

where $\delta {\omega }_{\text{Gauss}}=541 \text{ Hz}$ and $\delta {\omega }_{\text{Sinc}}=4000\text{ Hz}$ describe the width of the pulse in the frequency domain. The respective bandwidths were chosen for a field strength of 3 T, which is the clinical field strength which will later be applied (see below). Phantom measurements with a 10% PFOB fat–water emulsion were performed with both pulse shapes for all resonances using a single-channel Tx/Rx ^19^F solenoid coil. The signal intensities and chemical shift artifacts of the multi-resonance were compared.

### UTE sequence with Hadamard-encoding

**Fig. 3:**
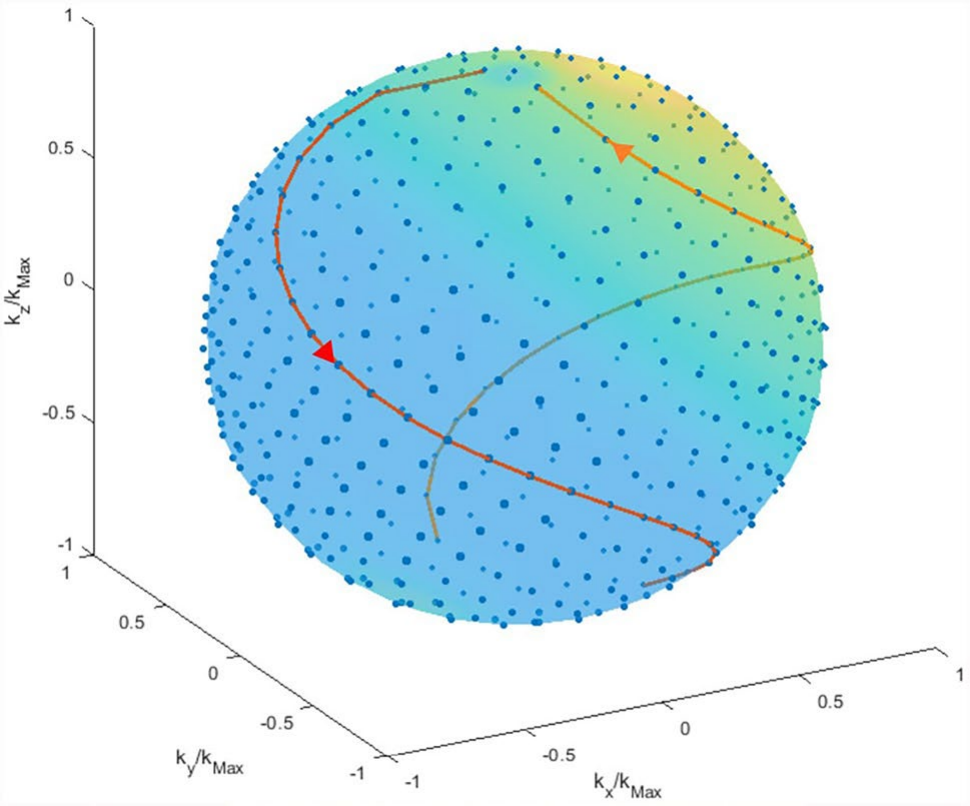
Phyllotaxis pattern of consecutively acquired spokes intersecting the unit sphere. The end-points of consecutive k-space spokes follow a spiral on the surface of a sphere with radius *k*_Max_. A spiral where the k_z_-component decreases with every spoke (red) is followed by a spiral with increasing k_z_-component (orange)

K-space data were acquired using a center-out radial trajectory with a spiral phyllotaxis pattern [26]. Here, all acquired spokes in k-space intersect a spherical spiral, where the azimuthal angle is rotated by the golden angle for consecutive points (Fig. 3). The spokes are acquired in an interleaved fashion starting with every *n*-th point, then rotating the starting point by a golden angle. For *n* being a Fibonacci number, consecutively acquired spokes are close to each other, reducing eddy current effects and improving gradient spoiling [27]. This approach was taken for two reasons: First, from FID measurements, we estimated T_2_* values for all resonances to be below 10 ms, so that an ultra-short echo time (UTE) sequence was chosen to maximize the signal. Second, to acquire images in the abdomen, the sequence needs to be robust to motion. While this is the case for radial imaging, the phyllotaxis trajectory ensures homogeneous sampling of k-space over small time scales compared to the total imaging time.

### PFOB nanoemulsion

A nanoemulsion consisting of 35% PFOB was used for the in vivo experiments. The PFOB nanoemulsion (PFOB-NE) was produced similar as described previously [12]: a buffer of sodium dihydrogen phosphate (0.24 g), disodium hydrogen phosphate (2.46 g), sodium chloride (1.15 g) in sterile water for injection (284.5 g) was mixed with 18.4 g Lipoid E 80SN (Lipoid GmbH, Ludwigshafen, Germany), 322 g perfluorooctyl bromide (PFOB) and 8.62 g 1-perfluoro-*n*-hexy-decane (both abcr GmbH, Karlsruhe, Germany). This mixture was pre-emulsified with a tip sonicator (Branson Ultrasonics Corporation) for 5 min in portions of 50 ml and then transferred to a CF1 homogenizer (Constant Systems Ltd., Daventry, UK) for 10 cycles of 1000 bar. Then, after renewed emulsification with the tip sonicator, the resulting emulsion was autoclaved for 15 min at 121 °C. The nanoparticles of the final emulsion had a typical mean of the number average size distribution of 170–190 nm measured by dynamic light scattering (Malvern Panalytical). The PFOB-NE was injected 3–4 days after production and was stored at 4 °C.

### *T*_1_ measurements

To optimize the flip angle for each resonance individually, *T*_1_ was determined in a 10% PFOB fat–water emulsion. To estimate *T*_1_, the flip angle of an FID MRS sequence (TE > 1.5 ms, dwell time = 50 µs) was varied (*α* = 0°–45°) in steps of 1° at a fixed TR = 30 ms, again using the single-channel Tx/Rx ^19^F solenoid coil. For RF excitation, a sinc pulse (duration: 3 ms, spectral width: 100 ppm) was used to ensure homogeneous excitation over the entire PFOB spectrum. Spectral boundaries for five resonances (CF_2_Br, CF_3_ and the three most prominent peaks $(\beta ,\delta +\epsilon ,\eta )$ from the CF_2_ multi-peak spectrum) were chosen, over which the signal strength was integrated for each flip angle. *T*_1_ was then estimated for each of these resonances using the FLASH signal equation [25].

### Phantom experiments

To test the validity of the method, phantom experiments were performed using the coil setup for the in vivo experiments, which consists of an eight-channel Tx loop coil array and an eight-channel Rx coil array containing two coils posterior and a six-channel anterior flexible array of coils [28]. The measurement was performed using the HE UTE sequence with TE = 1.7 ms, TR = 10 ms, readout bandwidth = 390 Hz/px, with 377 spirals and 26 spokes per spiral. The FWHM of the point-spread function was 15.7 mm in every direction, considering both regridding artifacts and T2*-decay, as well as the CF_2_ off-resonances. Regridding was performed using a voxel size of (5 mm)^3^. 8 Phantoms containing a 5% PFOB fat–water emulsion were measured ten times consecutively, resulting in a total acquisition time of 65:20 min:s. Due to the uneven distribution of noise in radial images, this allowed for a better noise estimation by creation of a standard deviation map [29]. SNR values of the reconstructed images for each peak and of the combined image were compared.

### Comparison with iterative deconvolution

We compared the HE UTE reconstruction with an iterative deconvolution as it is the most commonly used method for chemical shift artifact correction of PFOB, and can be applied to the same data set, allowing for a direct comparison. For this, we acquired an image of the same phantoms as before using the HE UTE sequence (TE = 1.7 ms, TR = 10 ms, readout bandwidth = 390 Hz/px, with 1597 spirals and 26 spokes per spiral) and measured an FID with the same bandwidth and TR with 500 averages. Then we applied an iterative sparse deconvolution algorithm to the signal for which each peak was excited with a positive sign, minimizing the function

7 $$x=\underset{\widetilde{x}}{\text{argmin}}{\Vert C\widetilde{x} -y\Vert }_{2}^{2}+\lambda {\Vert \widetilde{x}\Vert }_{1},$$

where *C* is the convolution matrix calculated from the FID signal, *y* the acquired data and $\lambda$ an arbitrary factor for LASSO (least absolute shrinkage and selection operator) regularization [18, 30]. $\lambda$ was chosen by trial and error, such that the final image yielded the maximum SNR without showing major artifacts. Reconstruction was done with Matlab (R2023a, The MathWorks Inc., Natick, USA) and took 2–4 h for each value of $\lambda$ using a GPU (NVIDIA GeForce RTX 2080 Super). SNR was measured for both methods, adjusted by a factor of 2 for the deconvolution method, as only ¼ of the data was used, and compared.

### In vivo experiments

One healthy juvenile pig (German landrace, 63 kg) was injected with a PFOB nanoemulsion (5 ml per kilogram body weight), and ^19^F-MRI scans were performed with the proposed HE UTE sequence 2, 4 and 7 days after the injection. To estimate the evolution of the ^19^F signal over time, the mean values of the acquired signals were compared in regions of the spleen that were equidistant from the coils in all measurements. Anesthesia was introduced and maintained as described previously [31]. The MRI protocol consisted of a *T*_1_ VIBE-Dixon sequence for ^1^H images, with TE = 1.29 ms, TR = 3.97 ms, readout bandwidth 1042 Hz/px and a matrix size of 260 × 320 per slice, 52 slices, voxel size 1.19 mm × 1.19 mm × 5 mm acquired in one breath hold through a brief interruption of mechanical ventilation. The HE UTE sequence to measure the ^19^F signal had the following acquisition parameters: TE = 1.7 ms, TR = 10 ms, readout bandwidth = 390 Hz/Px, with 1597 spirals and 26 spokes per spiral, resulting in a total acquisition time of 27:41 min:s. All measurements were performed on a clinical 3 T system (Prisma, Siemens Healthineers, Erlangen, Germany) and the ^19^F images were reconstructed using Kaiser–Bessel regridding in Matlab (R2023a, The MathWorks Inc., Natick, USA).

## Results

### *T*_1_ measurements

**Fig. 4:**
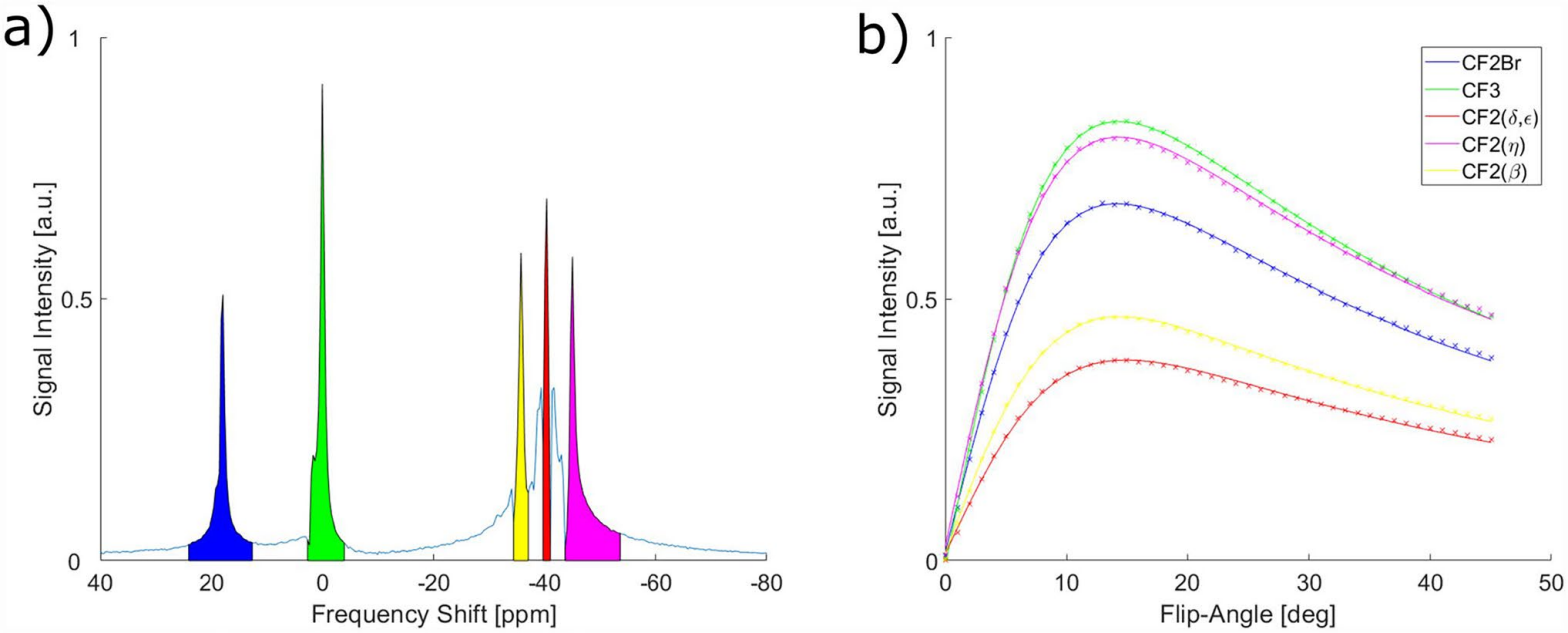
**a** Spectrum of perfluorooctyl bromide (PFOB), **b** measured FLASH signal curves of different resonances at TR = 10 ms

Figure 4a shows the PFOB spectrum together with the FLASH signal curves of the CF_3_ resonance, the CF_2_Br resonance and the most prominent resonances (*β*, *δ* + *ε*, *η*) of the CF_2_ multi-peak (Fig. 4b). *T*_1_ values of the different PFOB resonances were between 870 and 970 ms for all resonances (Table 1). Thus, at a TR of 10 ms as used in the imaging sequence, the Ernst angles differ by less than 0.5°. A variation of the flip angle by that amount changes the signal by only 0.3%. Therefore, all resonances were excited with the same flip angle (*α* = 8°) in future measurements.

**Table 1:** *T*_1_ values for different PFOB resonances together with their frequency range relative to the central resonance

Resonance	*T*_1_ [ms]	Frequency range [ppm]
CF_2_Br	966 ± 8	[12.6, 24.1]
CF_3_	949 ± 8	[− 4.0, 2.6]
CF_2_ (*β*)	874 ± 15	[− 37.0, − 34.3]
CF_2_ (*δ*, *ε*)	952 ± 12	[− 41.0, − 39.7]
CF_2_ (*η*)	940 ± 12	[− 53.6, − 43.6]

### RF pulse envelopes

**Fig. 5:**
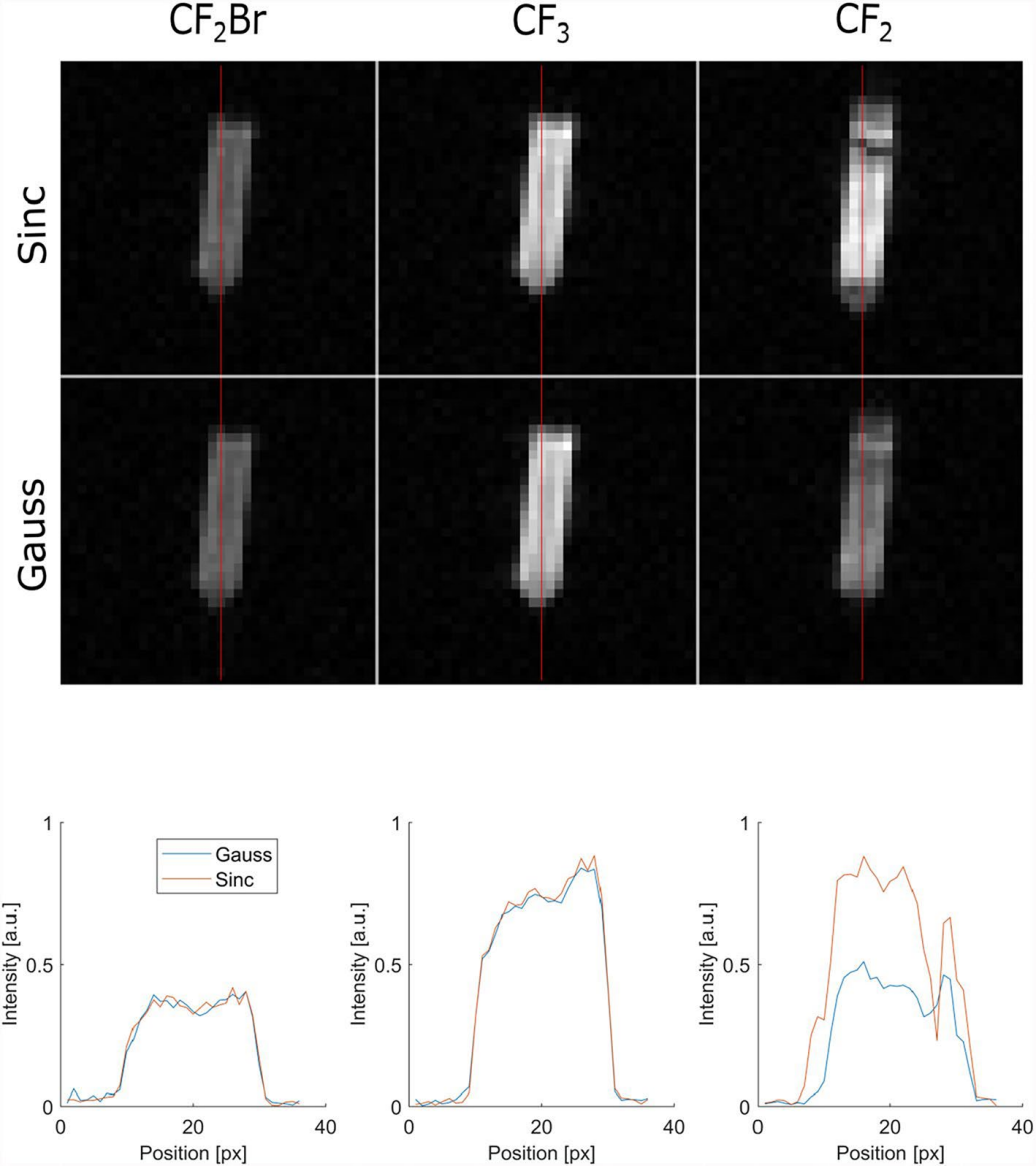
Comparison between excitation with Gaussian and sinc-shaped RF pulses for all PFOB resonances

A comparison between signals of Gaussian and sinc pulse envelopes is shown in Fig. 5. As expected, the SNR of the CF_2_ multi-peak was increased with the sinc pulse due to the more homogeneous excitation of its full spectrum leading to an SNR gain of 50%. Due to a broader excitation of the spectrum, a sinc envelope also increases chemical shift artifacts from these resonances. No significant difference was observed for the other resonances. Thus, for all further measurements, a Gaussian pulse was used for the CF_2_Br and CF_3_ resonances $(\delta {\omega }_{\text{Gauss}}=541 \text{ Hz})$ and a sinc pulse was used for the CF_2_ multi-peak $(\delta {\omega }_{\text{Sinc}}=4000 \text{ Hz})$.

### Phantom experiments

**Fig. 6:**
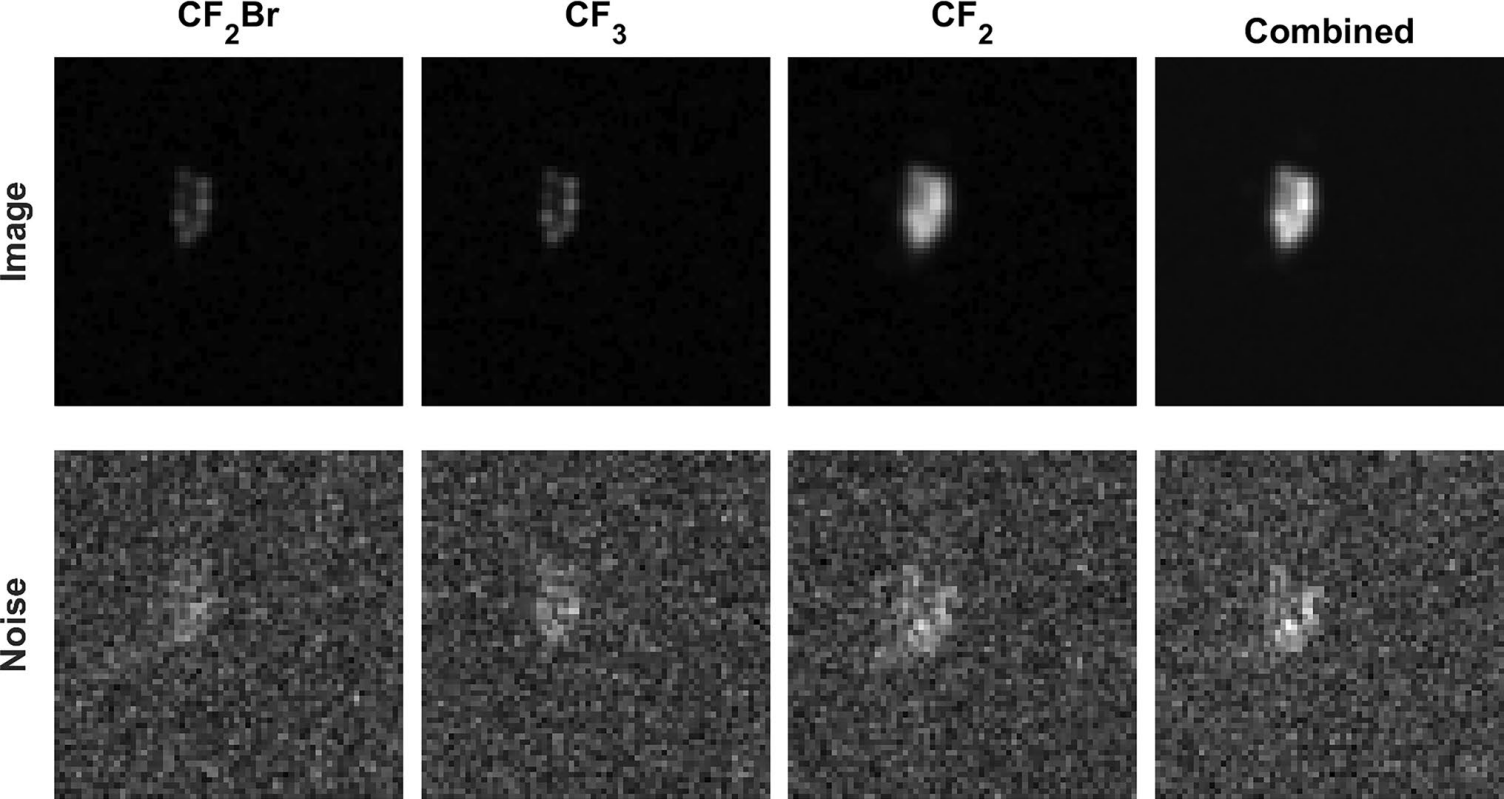
^19^F Phantom images acquired with the HE UTE sequence. Top: Comparison of individual reconstructions of each resonance with the combined image (mean of 10 averages). Bottom: Comparison of noise between each resonance and the combined image (Standard deviation of 10 averages)

Images and noise maps are seen in Fig. 6. SNR values for the single resonance images were SNR(CF_2_BR) = 55, SNR(CF_3_) = 65 and SNR(CF_2_) = 120 and for the combined image SNR(total) = 139. Thus, the SNR gain compared to the CF_2_ image was 15%.

### Comparison with iterative deconvolution

**Fig. 7:**
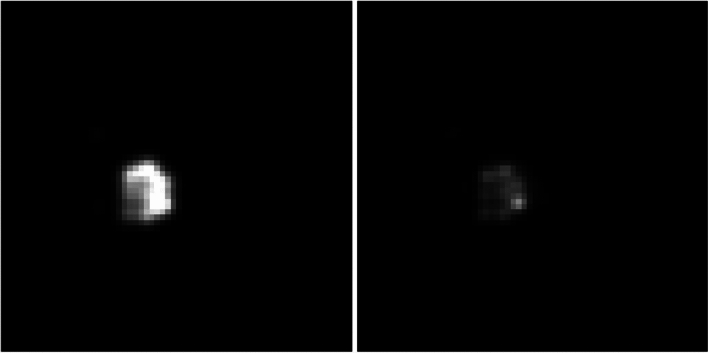
Image reconstructed with Hadamard-decoding (left) and iterative deconvolution (right)

Reconstruction of the data with both Hadamard-decoding and iterative deconvolution are shown in Fig. 7. The best reconstruction was found for $\lambda =3\cdot {10}^{-8}$. We measured an SNR of 297 for Hadamard-decoding and 133 for iterative deconvolution. The SNR ratio was $\frac{\text{SNR}\left(\text{HD}\right)}{2*\text{SNR}(\text{ID})}=1.11$ between both methods.

### In vivo results

**Fig. 8:**
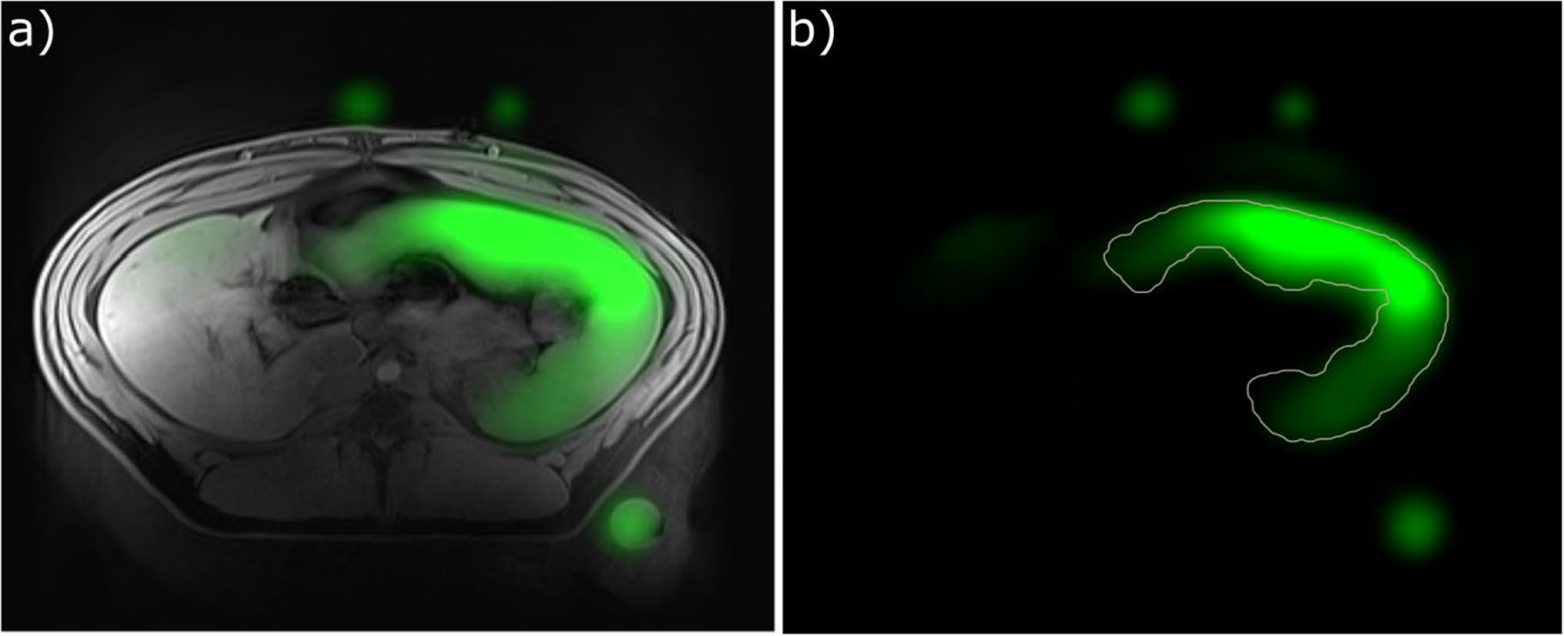
**a** Combined ^1^H (gray) and ^19^F (green) image of a porcine abdomen in vivo 2 days after PFOB-NE injection. **b** Outlines of a segmentation of the spleen together with the fluorine signal. The three green spots outside of the body of the pig are ^19^F marker vials. Due to blurring from the CF_2_ off-resonances, the edges appear less sharp in the ^19^F-signal

**Fig. 9:**
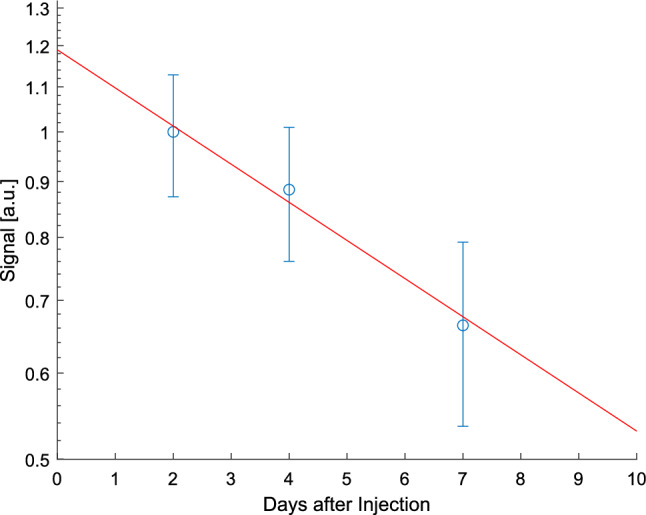
^19^F signal in the spleen at different times after injection on a logarithmic scale

Figure 8 shows the ^19^F signal in the spleen of a pig 2 days after injection of 5 ml/kg of PFOB-NE using the HE UTE ^19^F technique. A ^19^F signal with SNR over 100 was observed in the spleen, confirming the viability of the method of HE for this application. To quantify the temporal change of the PFOB concentration, the spleen was segmented on the ^1^H images. Figure 9 shows the average ^19^F signal intensity inside the spleen at 2, 4 and 7 days after injection on a logarithmic scale. By fitting the signal, the half-life of PFOB in the spleen is roughly estimated to be 9 ± 5 days. The adjusted R^2^ of the fit was 0.97 with a RMSE of 8% of the day 2 signal. Further PFOB signals were found in liver, bone marrow and subcutaneous fat in all measurements.

## Discussion and conclusion

In this work, a HE 3D UTE sequence was successfully implemented to image ^19^F PFOB in vivo. In phantom experiments, the *T*_1_ values of the PFOB resonances varied by only 3% so that a single flip angle could be used for all resonances. Exciting the CF_2_ resonances with a sinc pulse instead of a Gaussian pulse increased the SNR substantially, as the sinc pulse covers a larger spectral range and includes all CF_2_ resonances. With these optimizations, an SNR > 100 could be achieved in vivo which enabled the observation of the decreasing ^19^F signal in the spleen over a time-course of 7 days caused by the natural decay of monocytes.

Many ^19^F-MRI studies have been conducted so far in mice to image the spleen. For this, a range of different PFCs, such as PFOB [17, 21, 30], perfluorocrownether (PFCE) [16], FC-43 [14] or perfluoro(t-butylcyclohexane) (ABL-101) [32] were used and chemical shift artifact correction was performed with a variety of methods, for example iterative deconvolution [30] or spectroscopic imaging [21]. In this study a large animal model was used, and the chemical shift correction of the multi-peak ^19^F signal was performed by HE. The large animal model was chosen due to its geometric similarity to humans [33], as we want to use this method in studies of coronary artery interventions after myocardial infarction in the future [31, 34]. In general, a direct comparison of the SNR in this study with previous small animal studies is difficult due to the use of different RF coils, field strengths and voxel dimensions. Only few studies on pigs at a clinical 3 T system were performed which focused on imaging macrophages and other myeloid cells in the infarcted myocardium [11, 12]. So far, these studies used selective excitation of a single peak of the PFOB spectrum. Here, we have shown the viability of HE to image the whole PFOB spectrum at 3 T: HE enables a combination of the individual resonance signals to remove the chemical shift artifact, and it even allows to study the different resonances separately. With HE an increase in SNR by 15% was achieved over the excitation of a single resonance (CF_2_) and by 11% over using a time intensive iterative deconvolution approach, but the use of the composite HE RF pulses led to an increased TE, and thus to a slightly reduced SNR. Here, a TE = 1.7 ms was used, whereas a TE = 0.1 ms was possible for a single resonance. To further shorten the echo time in HE, asymmetric pulses could be applied which would lead to an estimated value of TE of about 0.2 ms which would further increase the ^19^F-MRI signal in a HE acquisition.

The HE signal shows a high ^19^F concentration in the spleen as seen in Fig. 7. ^19^F signal is also visible outside of the spleen, which is a consequence of the remaining uncorrected chemical shifts of the CF_2_ multi-peaks. This effect could be reduced either by exciting a narrower part of the CF_2_ spectrum, which in turn would lower the SNR, or using a deconvolution approach on top. A perfect deconvolution would remove all chemical shift artifacts; however, in practice deconvolution is a mathematically ill-posed problem that cannot be solved analytically and requires a computationally intensive reconstruction process. With the current sequence parameter settings, the blurring of the multi-peak CF_2_ signals is acceptable, as it extends about two pixels only. This could be reduced further by an increased pixel bandwidth. The HE method, however, is very simple as compared to a deconvolution approach, and it can be integrated directly into the image reconstruction so that ^19^F images are directly available after the image acquisition.

Over the course of the 30 min-long measurement breathing is expected to cause motion artifacts, which are not seen in underlying ^1^H images which were acquired in a breath hold. As ^19^F data are acquired with radial sampling, which is largely insensitive to periodic motion, these artifacts are expected to be small. Furthermore, they could be corrected using methods based on the low-frequency k-space data such as self-gating [35], which allows for sorting of the data into different phases of the breathing cycle at the cost of SNR.

A limitation of the longitudinal animal experiment is the comparison of the ^19^F signals acquired on subsequent days: the spleen’s position varied between measurement days, which can cause variations in its positioning within the Rx sensitivity profile. Nevertheless, the acquired data provide a good estimate of the temporal behavior of the injected PFOB, as they are integrated over the full volume of the spleen. Furthermore, the positioning of the animal was performed carefully to minimize re-positioning errors. All acquisitions show an SNR > 100 within the spleen, and, therefore, produce a relevant ^19^F signal.

In summary, using optimized flip angles and excitation pulses, HE ^19^F-MRI can be successfully applied to detect PFOB with high SNR in large animals. With this method, longitudinal studies might become feasible, for example to study the inflammatory reaction after acute cardiovascular events.

## Data Availability

Data will be provided upon reasonable request by the corresponding author.
